# Efficacy and safety of ketamine for post‐traumatic stress disorder: A systematic review with narrative synthesis and consideration of military and veteran populations

**DOI:** 10.1002/pcn5.70425

**Published:** 2026-10-07

**Authors:** Mary Ivasyutyn, Saajan Bhakta

**Affiliations:** ^1^ Department of Research Kansas Health Science University—Kansas College of Osteopathic Medicine (KansasCOM) Wichita Kansas USA

**Keywords:** ketamine, NMDA receptor antagonist, post‐traumatic stress disorder, PTSD, veterans

## Abstract

Post‐traumatic stress disorder (PTSD) is a debilitating disorder with limited pharmaceutical treatment options. Ketamine, a noncompetitive *N*‐methyl‐d‐aspartate (NMDA) receptor antagonist with rapid‐onset effects, has emerged as a potential treatment, particularly for patients who do not respond to first‐line therapies. This systematic review synthesizes the current evidence on the effectiveness and safety of ketamine for PTSD, with a focus on military and veteran populations. Eligible studies included adults with PTSD, who evaluated ketamine or esketamine, and reported efficacy and safety outcomes. Due to substantial clinical and methodological heterogeneity, a meta‐analysis was not performed. Eight studies (*N* = 383) were included, comprising five randomized‐controlled trials and three uncontrolled or observational studies. Most studies reported reductions in PTSD symptoms following ketamine administration. The largest effect size (*d*’ = 2.17; representing a sample size‐adjusted Cohen's *d*) was reported in a small uncontrolled open‐label study of 15 veterans, in which six ketamine infusions were associated with an 80% PTSD remission rate. This finding warrants caution, given the small sample and the absence of a control group. Dissociative and hemodynamic adverse effects were frequently observed during infusion, but typically resolved afterward. No study reported cognitive decline associated with repeated ketamine administration. Current evidence suggests that ketamine may be associated with short‐term reductions in PTSD symptoms in some populations; however, findings remain preliminary due to small samples, heterogeneous designs, brief follow‐up, and inconsistent safety reporting.

## INTRODUCTION

Post‐traumatic stress disorder (PTSD) is a serious psychiatric condition that develops after experiencing or witnessing life‐threatening events, such as actual or threatened death, serious injury, or sexual violence. The key symptomatic features of PTSD include re‐experiencing, avoidance, persistent cognition and mood changes, and heightened arousal.^1^ Globally, the prevalence of PTSD is estimated to be approximately 3.9%. However, this rate is markedly higher among individuals exposed to high‐intensity trauma, including combat, sexual assault, and natural disasters.^2^ Accordingly, PTSD is associated with significant disability, reduced quality of life, elevated suicide risk, and frequent comorbidities, such as major depressive disorder, substance use disorders, and chronic pain.^3^

Despite its prevalence, pharmacological treatment options for PTSD remain limited. Currently, only two selective serotonin reuptake inhibitors (SSRIs)—sertraline and paroxetine—have been approved by the US Food and Drug Administration (FDA) for PTSD management.^4^, ^5^ Although they have been reported to provide symptom relief for a subset of patients, many fail to achieve remission, with clinical improvement taking weeks or longer.^6^ Moreover, SSRIs appear to be particularly limited in addressing symptoms such as hyperarousal and re‐experiencing.^4^, ^7^ Consequently, more effective and rapid pharmacotherapy, particularly for acute or treatment‐resistant cases, is urgently needed.^4^

Ketamine, a noncompetitive *N*‐methyl‐d‐aspartate (NMDA) receptor antagonist, has recently been explored as a promising, rapid‐acting drug for therapy‐resistant psychiatric disorders.^8^ Initially developed as an anesthetic drug, ketamine has demonstrated efficacy as an antidepressant for the management of various mood disorders.^9^, ^10^, ^11^ The FDA approval of intranasal esketamine (S‐ketamine; Spravato) for TRD in 2019 has not only supported the glutamatergic system as a therapeutic target but has also paved the way for further research into ketamine.^11^

The use of ketamine in PTSD is also supported by neurobiological evidence, with NMDA receptor signaling involved in memory acquisition and fear extinction. Moreover, studies report the association between maladaptive fear learning seen in cases of PTSD with changes in NMDA receptor signaling in the amygdala, the prefrontal cortex, and the hippocampus.^12^, ^13^ Therefore, NMDA receptor blockade by ketamine, together with subsequent mTOR pathway activation, synaptogenesis, and increased brain‐derived neurotrophic factor (BDNF) levels, may facilitate the reconsolidation or extinction of fear memories while helping restore dysfunctional connectivity between the amygdala and the prefrontal cortex.^14^ Importantly, preliminary clinical studies have reported potential therapeutic effects of ketamine in PTSD populations,^15^ although the evidence remains limited.

Military personnel and veterans represent particularly important populations in PTSD research because of their high exposure to combat‐related trauma, substantial burden of chronic and treatment‐resistant symptoms, and frequent psychiatric and medical comorbidities. Combat‐related PTSD is frequently characterized by medication resistance and often complicated by comorbidities such as traumatic brain injury (TBI), chronic pain, moral injury, and heightened suicide risk.^16^ The prevalence of post‐deployment PTSD among veterans has been reported to range from 4% to 30%,^17^ and the response to pharmacotherapy in this population is generally less favorable than in civilians.^18^ As a result, several research centers investigated the potential role of ketamine in treating PTSD among veterans, although the available findings remain preliminary and warrant further study.^19^

Previous systematic reviews and partial meta‐analyses have evaluated ketamine for PTSD; however, the available evidence remains limited by small sample sizes, heterogeneous study designs and treatment protocols, inconsistent safety reporting, and limited consideration of military and veteran populations.^20^ Rather than providing an additional pooled efficacy estimate, the present review was designed to provide an updated structured narrative synthesis of the clinical evidence, with particular attention to methodological differences that affect interpretation across studies.

Specifically, this review aimed to (1) summarize evidence on the effectiveness of ketamine in alleviating PTSD symptoms across randomized and nonrandomized clinical studies; (2) evaluate available safety and tolerability data, including dissociative and psychotomimetic effects, hemodynamic changes, cognitive outcomes, and the limited evidence regarding abuse liability and dependence; and (3) descriptively examine findings from military and veteran populations without implying a formal subgroup analysis. In contrast to previous reviews, the present review therefore integrates efficacy, safety, and methodological considerations, including functional unblinding, incomplete adverse‐event reporting, cognitive outcomes, and abuse liability, while providing a focused descriptive assessment of the evidence from military and veteran populations.

## METHODS

### Protocol registration and reporting

This systematic review was conducted and reported in accordance with the 2020 Preferred Reporting Items for Systematic Reviews and Meta‐Analyses (PRISMA 2020) standards.^21^ A PRISMA 2020 checklist is provided as an additional document for this submission. The review was registered in the PROSPERO database on May 20, 2026 (registration number: CRD420261401140). The eligibility criteria, search strategy, and planned analyses were not modified after initiation of the review or following registration. The delayed registration is acknowledged as a methodological limitation.

### Eligibility criteria

Studies were selected using a pre‐specified PICOS framework.^22^

#### Population (P)

Adults aged 18 years or older with a verified diagnosis of PTSD following set criteria (DSM‐IV, DSM‐5, ICD‐10, or ICD‐11). No restrictions were imposed on trauma type. Military personnel and veterans were identified a priori as a population of specific interest for descriptive analysis.

#### Intervention (I)

Ketamine or esketamine (S‐enantiomer) in any form, dosage, route of administration (IV, IM, nasal, or oral), and treatment duration. Trials that studied ketamine monotherapy or ketamine in conjunction with other drug therapies were included.

#### Comparator (C)

Saline or placebo infusion, pharmacologically active control arm (such as midazolam), pretreatment baseline data collection (for single‐arm and open‐label studies), or best medical care (SOC).

#### Outcomes (O)

The main outcome measures included changes in PTSD symptom severity, assessed using standardized and validated tools such as the CAPS‐5 and PCL‐5 for the DSM‐5, the Impact of Event Scale‐Revised (IES‐R), and the Davidson Trauma Scale. Response and remission rates were recorded when available. Other secondary measures included changes in depression, anxiety, suicide risk, pain symptoms, functional impairment, quality of life, and safety endpoints (dissociation using Clinician‐Administered Dissociative States Scale [CADSS], psychotomimetic effects, hemodynamics, adverse events, SAEs, and withdrawals due to intolerance).

#### Study Design (S)

Randomized‐controlled trials, non‐randomized control trials, prospective open‐label trials, retrospective observational cohort studies, and case series with at least five subjects were included. Single‐case studies, animal studies, *in vitro* experiments, reviews, editorials, correspondence studies, and conference papers with no original patient data were excluded.

### Information sources and search strategy

An extensive literature search was conducted across five online bibliographic databases, including PubMed, Cochrane, ScienceDirect, Web of Science, and Google Scholar, from the earliest available records in each database to the date of the last search, as shown in Table 1. Google Scholar was used as a supplementary search source to identify additional potentially relevant studies. Because Google Scholar retrieves a large ranked set of results with decreasing relevance and limited reproducibility, screening was restricted to the first 100 results sorted by relevance.

**Table 1:** Search strategy by database.

Database	Search string	Records retrieved
PubMed	(PTSD OR “post‐traumatic stress disorder”) AND (ketamine OR esketamine) AND (treatment OR therapy OR intervention)	204
Web of Science	(“post‐traumatic stress disorder” OR “posttraumatic stress disorder” OR PTSD) AND (ketamine OR esketamine OR “S‐ketamine” OR Spravato) AND (“randomized controlled trial” OR “clinical trial” OR “open‐label” OR cohort OR efficacy OR safety)	177
Cochrane	(“post‐traumatic stress disorder” OR “post traumatic stress disorder” OR PTSD) AND (ketamine OR esketamine) AND (random* OR “randomized controlled trial” OR placebo OR trial OR “open label”)	89
ScienceDirect	(“post‐traumatic stress disorder” OR PTSD) AND (ketamine OR esketamine) AND (efficacy OR safety OR outcomes OR clinical trial OR randomized) (Note: military and veteran terms used as subgroup filter during screening only, not as mandatory search terms)	617
Google Scholar	ketamine “post‐traumatic stress disorder” efficacy safety clinical trial (first 100 results reviewed)	100
TOTAL		1,187

Terms relating to the key concepts used in combination with Boolean operators were (1) the condition (“post‐traumatic stress disorder,” “PTSD,” “trauma‐related disorder”); (2) the intervention (“ketamine,” “esketamine,” “S‐ketamine,” “Spravato,” “NMDA receptor antagonist,” “ketamine infusion”); and (3) study design (“randomized controlled trial,” “clinical trial,” “open label,” “cohort”). Outcome terms (“efficacy,” “remission”) and population qualifiers (“military,” “veterans”) were used only as filters at the screening stage rather than as search terms, as they would have potentially excluded articles about the civilian use of ketamine to treat PTSD. Search terms included controlled vocabulary where available, such as MeSH terms in PubMed, along with database‐specific keyword searches. The full search strings for both databases are listed in Table 1.

### Study selection

The selected references were imported into Covidence systematic review software (Veritas Health Innovation, Melbourne, Australia), and duplicates were removed. The current systematic review was performed by only one reviewer, which is considered a drawback of the review process that could have led to bias during the selection process. Title and abstract screening were performed based on the inclusion/exclusion criteria, followed by a full‐text evaluation of all potentially eligible studies. Reasons for the exclusion of articles from the full text are provided. The included‐study decisions, extracted information, risk‐of‐bias assessments, and manuscript tables were subsequently subjected to supervisory review and verification by the senior author.

### Data extraction

Data were extracted using a standardized, pilot‐tested data extraction tool that provided information about the included studies, such as study characteristics (author, year of publication, country, study design, setting, period of recruitment, sample size, and funding source), participant details (mean age, sex, diagnostic criteria, trauma type, PTSD chronicity/severity, comorbidity, and veteran status), intervention (ketamine formulation, administration route, dose, and number of doses), control intervention, and outcomes (outcome measures, time points, raw scores, change scores, effect sizes, confidence intervals, response, and effects).

### Data synthesis

Clinical and methodological heterogeneity was anticipated across the included studies, including differences in patient populations, ketamine formulation and route of administration, treatment schedules, comparator groups, study design, outcome measures, and assessment timing. Quantitative pooling was considered both across the randomized‐controlled trials and within narrower clinically comparable subsets. However, an overall meta‐analysis was not considered clinically interpretable because the trials differed substantially in treatment regimen, including single versus repeated administration, intravenous versus intramuscular routes, doses ranging from 0.2 to 1.0 mg/kg, comparator type, study design, PTSD outcome measures, and follow‐up schedules. Single‐dose studies generally assessed outcomes within hours or days, whereas repeated‐dose studies evaluated symptom changes after six or eight infusions delivered over two to four weeks.

A restricted synthesis of Feder et al.^15^ and Dadabayev et al.^23^ was specifically considered because both studies administered a single intravenous ketamine dose of 0.5 mg/kg and assessed PTSD symptoms using the IES‐R at approximately 24 h. However, Dadabayev et al. did not report arm‐specific 24‐h IES‐R means and standard deviation, change scores with corresponding variance, or a treatment‐effect estimate with an associated standard error or confidence interval for the PTSD subgroup. Therefore, a compatible treatment effect could not be calculated reliably from the published data.

The two studies also differed clinically. Feder et al. compared ketamine with midazolam in adults with chronic PTSD, while Dadabayev et al. compared ketamine with ketorolac in patients with comorbid PTSD and chronic pain. Because these comparators differ in psychoactive, masking, and analgesic properties, pooling would combine clinically distinct comparator‐defined effects. Given these clinical and methodological differences, together with incompatible outcome reporting, a quantitative synthesis was not considered sufficiently reliable or clinically interpretable.

The results were therefore synthesized narratively in accordance with the Synthesis Without Meta‐Analysis (SWiM) guidelines. Where individual studies reported standardized mean difference (SMD) or Cohen's *d* statistics, these were presented on a per‐study basis to describe individual study effects rather than pooled estimates. Accordingly, the prespecified descriptive subgroup categories included (1) military or veteran versus civilian populations; (2) route of ketamine administration; (3) administration schedule; (4) number of treatment sessions; (5) concomitant psychotherapy; and (6) study design.

### Quality assessment

The quality of the methodology and risk of bias were evaluated using design‐specific instruments. The new Cochrane Risk of Bias tool version 2 (RoB 2) was used in randomized‐controlled trials (RCTs) in five dimensions: randomization process, performance of interventions as planned, incomplete outcome data, outcome measurements, and reporting of results.^24^ Open‐label and pre‐post observational studies were evaluated using the National Institutes of Health Quality Assessment Tool for Before‐After (pre‐post) Studies. Case series studies were evaluated using the NIH Quality Assessment Tool for Cases.^25^ The Newcastle–Ottawa Scale (NOS) was used to assess the quality of non‐randomized cohort and case–control studies by examining the selection, comparability, and outcome domains.^26^ Studies involving active control groups without complete randomization selected elements of the RoB 2 and NIH pre‐ and post‐study assessment criteria. Overall, judgments were classified into three categories: good, fair, and poor. The findings are depicted in Figures 2 and 3, and Tables 2, 3.

**Table 2:** NIH quality assessment—Case series.

Study	Q1: Research question clear?	Q2: Study population defined?	Q3: Participation rate ≥ 50%?	Q4: Same/similar population?	Q5: Sample size justification?	Q6: Exposure measured before outcome?	Q7: Adequate timeframe?	Q8: Different exposure levels examined?	Q9: Exposure measures valid?	Q10: Exposure assessed >once?	Q11: Outcome measures valid?	Q12: Outcome assessors blinded?	Q13: Follow‐up ≤ 20% lost?	Q14: Confounders adjusted?	Overall rating
Artin et al. 2022	Yes	Yes	No	Cannot determine	Yes	No	Cannot determine	No	Cannot determine	Yes	No	Yes	No	No	Fair

**Table 3:** Risk of bias assessment—Randomized‐controlled trials (parallel‐group and crossover designs) using RoB 2.

Author, year	D1: randomization	D2: deviations from intervention	D3: missing outcome data	D4: outcome measurement	D5: selection of reported result	Overall risk of bias
Feder et al.^15^	Low	Low	Some concerns	Some concerns	Low	Some concerns
Feder et al.^27^	Low	Low	Some concerns	Some concerns	Low	Some concerns
Abdallah et al.^28^	Low	Low	Some concerns	Some concerns	Low	Some concerns
Beaglehole et al.^29^	Low	Low	Some concerns	High	Low	High
Dadabayev et al.^23^	Low	Low	Some concerns	Some concerns	Low	Some concerns

### Publication bias

Formal tests for publication bias, such as funnel plot asymmetry and Egger's test, generally require at least ten studies to provide meaningful results. Given the small number of included studies and the absence of a meta‐analysis, a formal assessment of publication bias was not performed. It was rather evaluated by checking the registration status of the studies, when such information was provided, and using the selective outcome‐reporting item of the RoB 2 instrument. Therefore, the non‐publication of small, negative, or neutral studies cannot be ruled out.

## RESULTS

### Study selection

Database searches revealed 1187 manuscripts from the five selected electronic databases: ScienceDirect (*n* = 617), PubMed (*n* = 204), Cochrane (*n* = 89), Web of Science (*n* = 177), and Google Scholar (*n* = 100). After removing duplicates, 976 manuscripts were evaluated based on titles and abstracts. In total, 943 manuscripts were excluded because they failed to satisfy one of the specified inclusion criteria (lack of verified PTSD diagnosis; ketamine administered solely as an anesthetic, analgesic, or treatment for conditions other than mental illnesses; or an inappropriate study design). Full‐text evaluation was conducted on 33 records. Among these, 25 additional articles were excluded for the following reasons: did not meet eligibility criteria (*n* = 15), had an ineligible study design (n = 6), did not report outcome of interest (*n* = 3), or represented a secondary publication of an already included study cohort (*n* = 1). Eight studies reported in eight documents satisfied all the inclusion criteria and were included in the final review process. The study selection process is summarized in Figure 1.

**Figure 1:**
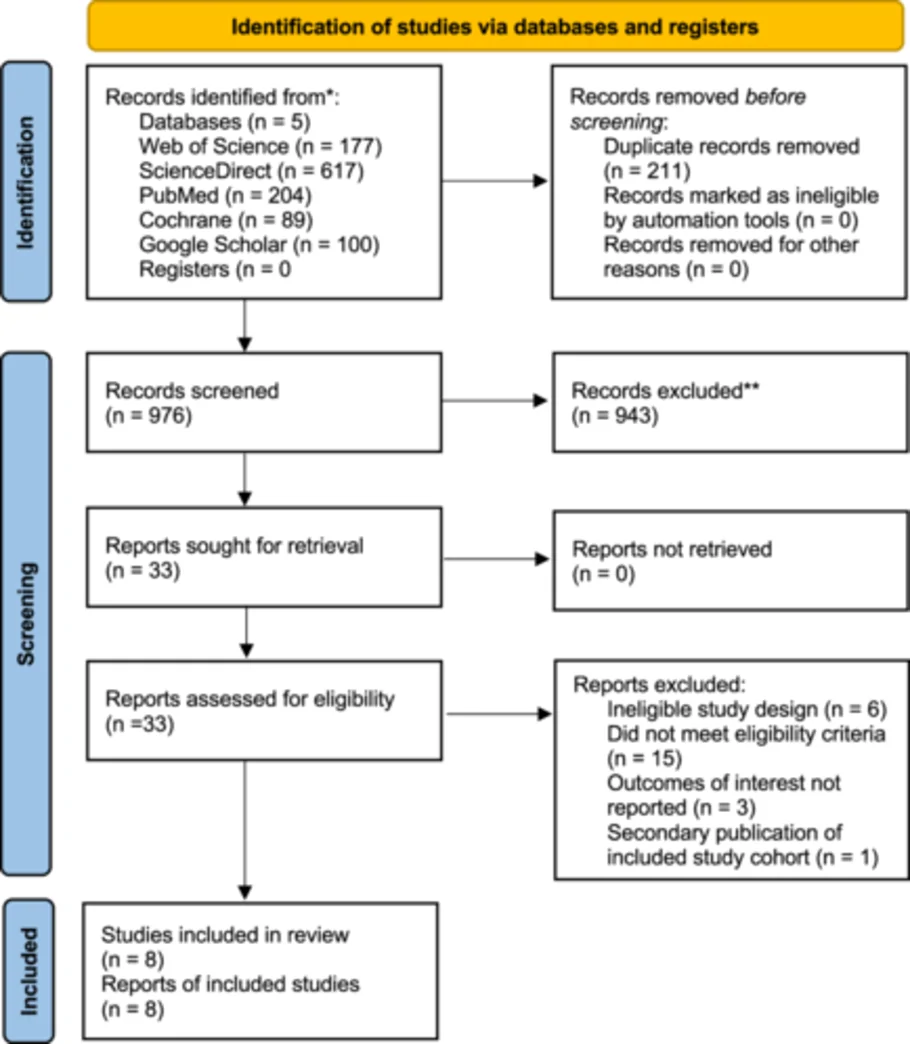
PRISMA flow diagram for study selection.

### Study characteristics and participant profiles

All eight included studies were original clinical investigations reporting patient‐level efficacy and/or safety outcomes following ketamine or esketamine administration for PTSD. These studies, published between 2014 and 2025, included a combined sample of approximately 383 participants. The patients were from various clinical sites, including university hospitals, Veterans Affairs outpatient departments, and military healthcare clinics. The corpus encompassed a range of study designs: five randomized‐controlled studies, including two randomized crossover trials and three parallel‐group trials; one open‐label single‐arm pre–post study; one prospective pre–post observational study; and one retrospective case series. Table 4 presents the details from each study. Half of the included studies were conducted exclusively or predominantly on veterans, whereas the remaining half focused primarily on civilian participants. Across all eight studies, the majority of participants were male, with the proportion ranging from approximately 66% to 77% in military veteran cohorts. PTSD diagnoses were established using either the DSM‐IV or DSM‐5 criteria in all studies. Baseline symptom severity was noted to be uniformly high, as reflected by elevated mean scores on the CAPS‐5, PCL‐5, and IES‐R. Major depressive disorder was the most frequently reported psychiatric comorbidity, followed by anxiety and substance use disorders.

**Table 4:** Characteristics of the included studies.

Author, design	Country/*N*	Population	PTSD criteria	Ketamine: route, dose, frequency	Comparator and follow‐up	Key efficacy findings
Abdallah et al.^28^ (DB RCT, multicentre)	USA; *N* = 158 (104/54)	Veterans and active‐duty military and veterans	DSM‐5 (CAPS‐5)	IV 0.2 or 0.5 mg/kg; 8 infusions over 4 weeks	Placebo (saline); 4 weeks post‐treatment	No significant overall dose‐related effect on PCL‐5/CAPS‐5; 0.2 mg/kg arm showed PCL‐5 reduction vs. placebo at week 4 (MD = 15.3; *p* = 0.03)
Albott et al.^30^ (Open‐label, single‐arm)	USA; *N* = 15 (15/0)	military veterans	DSM‐5	IV 0.5 mg/kg; 6 infusions over 12 days (Mon/Wed/Fri)	None (baseline); 8 weeks post‐final infusion	Significant improvement in PTSD and depression symptoms; no neurocognitive deterioration
Artin et al.^31^ (Retrospective case series)	USA (VA); *N* = 35 (35/0)	military veterans (VA)	DSM‐5	Intranasal esketamine 56 mg (flexible 28–84 mg); twice weekly ×4 weeks, then weekly	None (baseline); 4–16 treatments	PCL‐5 decreased by 15.5 points (54.8 → 39.3; *p* < 2e−7); 46% achieved ≥30% reduction
Beaglehole et al.^29^ (Randomized double‐blind psychoactive‐controlled crossover trial)	New Zealand; *N* = 33 (26/7)	community adults with treatment‐refractory PTSD; many with comorbid TRD	DSM‐5 PTSD; MINI interview; IESR ≥ 33	IM racemic ketamine 0.5 or 1.0 mg/kg; single‐dose injections across 3 crossover sessions ≥1 week apart	IM fentanyl 50 μg; follow‐up at 1 h, 2 h, 24 h, and 7 days	dose‐related IES‐R reduction vs. fentanyl; 81% response rate (> 50% IES‐R reduction) at 24 h with 1.0 mg/kg
Dadabayev et al.^23^ (DB RCT, active comparator)	USA (VA); *N* = 41 (21/20)	Military and veterans	DSM‐5 (CAPS‐5)	IV 0.5 mg/kg; single infusion over 40 minutes	Ketorolac 15 mg IV; 7 days	both groups showed comparable IES‐R reduction; no significant between‐group difference; greater pain relief in the PTSD + chronic pain subgroup (VAS − 30.61 vs. −14.28; *p* < 0.001)
Feder et al.^15^ (Randomized double‐blind crossover trial)	USA; *N* = 41 (22/19)	Adults aged 18–55 years with chronic PTSD related to multiple trauma exposures	DSM‐IV‐TR PTSD diagnosed using SCID; CAPS ≥ 50	IV ketamine 0.5 mg/kg; single infusion over 40 min	IV midazolam 0.045 mg/kg (active placebo); crossover design with infusions 2 weeks apart; follow‐up at 24 h, 48 h, 72 h, and 7 days	Significant IES‐R reduction vs midazolam at 24 h (MD = 12.7; *p* = 0.02); improvements across symptom clusters; transient dissociation
Feder et al.^27^ (Randomized double‐blind parallel‐arm controlled trial)	USA; *N* = 30 (15/15)	Adults aged 18–70 years with chronic PTSD; majority female; severe chronic PTSD (mean duration 14.9 years)	DSM‐5 PTSD diagnosed using SCID‐5 and CAPS‐5; CAPS‐5 ≥ 30	IV ketamine 0.5 mg/kg; 6 infusions over 2 consecutive weeks (approximately 3 infusions/week)	IV midazolam 0.045 mg/kg (psychoactive placebo); primary follow‐up at 2 weeks, responders followed weekly up to 4 weeks, and then monthly up to 6 months	Ketamine produced greater reduction in CAPS‐5 PTSD severity scores versus midazolam at week 1 and week 2 (difference at Week 2: −11.88 points; *d* = 1.13). Response rates were 67% with ketamine versus 20% with midazolam. Median time to relapse 27.5 days
Ross et al.^32^ (Prospective pre‐post)	USA; *N* = 30 (30/0)	US combat veterans	Not specified	IV high dose; 6 infusions (dose not reported)	None (baseline); baseline to post‐6th infusion	PCL‐5 reduced by 25 points (56.2 → 31.3; *p* < 0.001); PHQ‐9 reduced by 9.4 points (18.9 → 9.5; *p* < 0.001)

The predominant intervention was intravenous administration of racemic ketamine at a dose of 0.5 mg/kg delivered over 40 min, though notable variation existed across studies. While some trials compared multiple intravenous dose levels, others explored intranasal esketamine administration across a flexible dosing range. Treatment duration also varied considerably, ranging from a single session to six infusions administered over 12 days, with the longest regimen comprising up to eight infusions delivered over four weeks. The sample sizes were consistently modest throughout the evidence base, ranging from 15 to 158 participants.

### Risk of bias and methodological quality

A summary of the quality assessment findings is presented in Tables 3, 4, 5, and Figures 2 and 3. Five randomized‐controlled studies, including both parallel‐group and crossover randomized designs, were rated as having “some concerns” for overall risk of bias across most RoB 2 domains. The principal area of concern was the difficulty of blinding participants and study personnel, as ketamine's pronounced dissociative and psychotomimetic effects can make concealment of treatment allocation particularly challenging. Similarly, the pilot randomized and quasi‐experimental controlled studies received “some concerns” ratings for overall risk of bias, with the exception of Beaglehole et al.,^29^ which was rated as high risk of bias due to concerns in the measurement of outcomes domain. Among the uncontrolled studies evaluated using the NIH Quality Assessment Tool for Before–After Studies, two received overall ratings of fair, with limitations attributable to small sample sizes, absence of control conditions, and uncertainty regarding loss to follow‐up. The retrospective case series, appraised using the NIH Case Series tool, also received a fair rating, with nonconsecutive sampling and incomplete follow‐up identified as the primary weaknesses. Importantly, no study was assigned a poor overall rating using the NIH quality assessment tools, and all included articles used validated standardized measures of PTSD symptom severity.

**Table 5:** NIH quality assessment—Before–after (pre‐post) studies.

Study	Q1: Study question clear?	Q2: Eligibility criteria described?	Q3: Representative sample?	Q4: Eligible participants enrolled?	Q5: Sample size adequate?	Q6: Intervention clearly described?	Q7: Outcome measures valid?	Q8: Outcome assessors blinded?	Q9: Follow‐up ≤ 20% lost?	Q10: Pre‐to‐post statistics?	Q11: Multiple pre/post time points?	Q12: Group‐level analysis?	Overall rating
Albott et al.^30^	Yes	Yes	Yes	Yes	No	Yes	Yes	No	Yes	Yes	Yes	Yes	Fair
Ross et al.^32^	Yes	Yes	Yes	Yes	No	Yes	Yes	No	Cannot determine	Yes	Cannot determine	Yes	Fair

**Figure 2:**
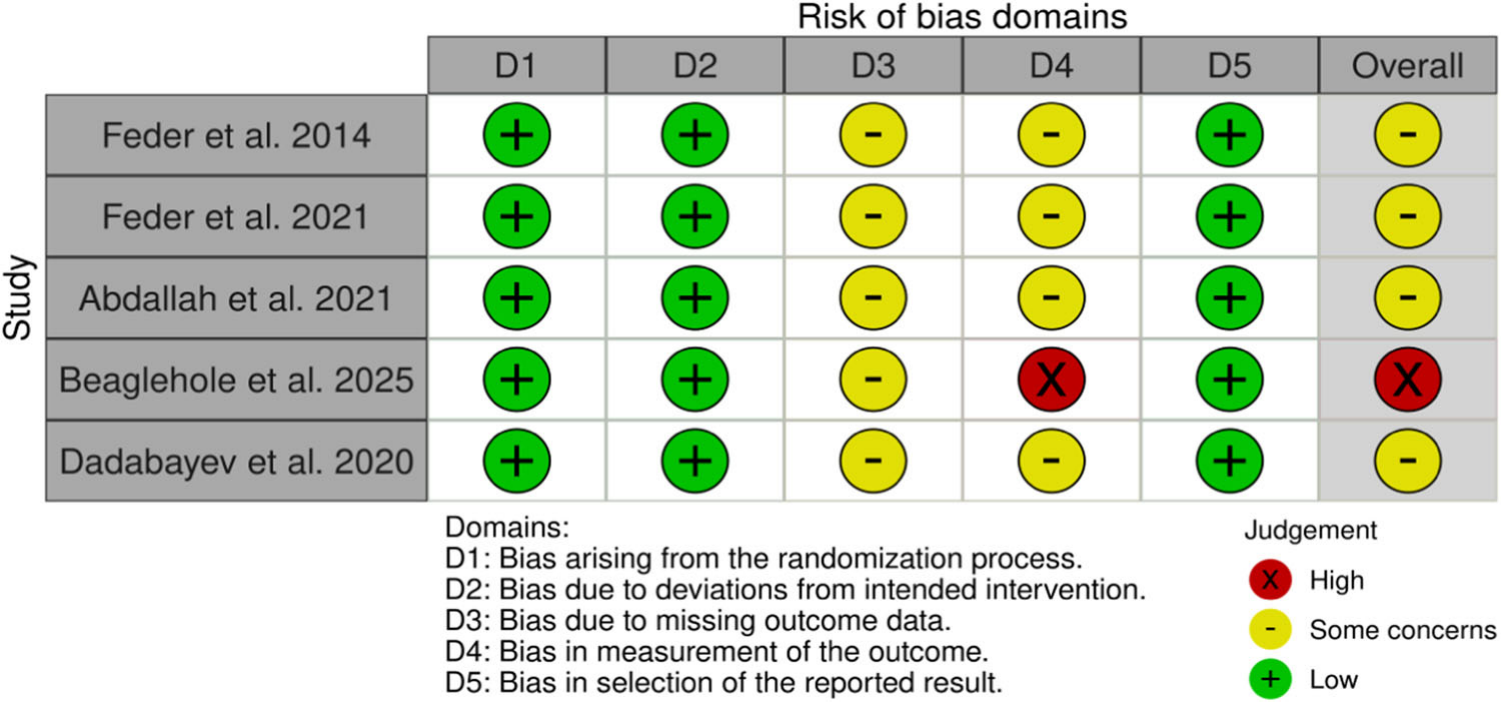
Traffic plot for risk of bias and quality assessment results.

**Figure 3:**
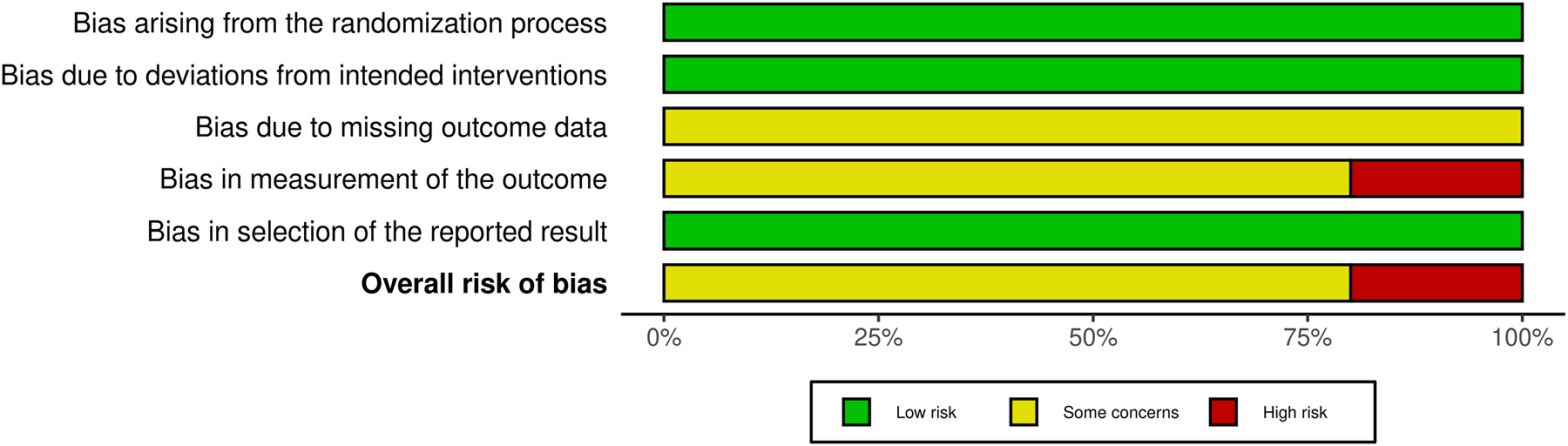
Summary plot of the outcomes of the critical appraisal of the studies.

### PTSD symptom outcomes

The eight studies all reported changes in the severity of PTSD symptoms after ketamine or esketamine administration. Most studies indicated symptomatic improvement, although the magnitude of the effect, statistical significance, and durability of the responses varied considerably. Among the randomized‐controlled trials, the largest study by Abdallah et al.^28^ (*n* = 158, predominantly military personnel) observed no clear dose–response relationship for PTSD symptom reduction based on both primary (PCL‐5) and secondary (CAPS‐5) outcome measures. Notably, a post hoc subgroup analysis demonstrated that participants receiving the lower dose (0.2 mg/kg) experienced a statistically significant improvement in PCL‐5 scores at four weeks relative to placebo (mean difference = 15.3 ± 5.8; adjusted *p* = 0.03). Among the non‐controlled trials, an open‐label cohort study by Albott et al.,^30^ which recruited 15 treatment‐resistant military veterans at one VA clinic each, revealed large pre‐ to post‐decreases in the severity of PTSD symptoms following six infusions of ketamine administered intravenously over 12 days. Additionally, there was a marked improvement in the PCL‐5 and CAPS‐5 scores, with statistical significance attained between baseline and Day 1 (*t* = 2.33, *p* = 0.03), and between baseline and Day 7 (*t* = 2.93, *p* < 0.01). These results were obtained from a single study with 15 participants who did not have a control group and, as such, should be interpreted with caution.

A prospective observational study by Ross et al.^32^ demonstrated a substantial reduction in PTSD symptom severity among 30 US combat veterans who received six 1‐h high‐dose intravenous ketamine infusions. The mean PCL‐5 scores decreased from 56.2 at baseline to 31.3 post‐treatment (*p* < 0.001). Similarly, in a VA case series of patients receiving intranasal esketamine (*N* = 35), the mean PCL‐5 score decreased by 15.5 points: from 54.8 to 39.3 (*p* < 2 × 10^−7^). Particularly, 46% of individuals achieved more than a 30% reduction in symptoms, a threshold generally considered clinically meaningful.^31^ Although ketamine and esketamine demonstrate therapeutic potential for alleviating PTSD symptoms, treatment responses are heterogeneous and may be shaped by comorbidities. For example, the double‐blind trial by Dadabayev et al.,^23^ which enrolled 41 veterans living with both PTSD and chronic pain, found that a single infusion of either ketamine 0.5 mg/kg or ketorolac 15 mg showed similar reductions in IES‐R scores on both days one and seven following the infusion. However, no significant difference was recorded in the degree of improvement in IES‐R scores between the two groups.

Dose‐dependent effects of ketamine have also been reported, with an intramuscular dose of 1.0 mg/kg producing the longest‐lasting symptom relief, as shown by Beaglehole et al.^29^ Moreover, 81% of participants experienced at least a 50% decrease in IES‐R scores within 24 hours.^29^ In another randomized‐controlled trial by Feder et al.,^15^ even a single 0.5 mg/kg i.v. ketamine infusion was shown to reduce PTSD symptoms at 24 hours compared to midazolam, an active control.^15^ Additionally, a more recent RCT by Feder et al.^27^ (*n* = 30, adults with chronic PTSD) demonstrated that, when ketamine was administered repeatedly over 2 weeks (six IV infusions of 0.5 mg/kg), participants experienced greater improvements, a higher response rate (67% vs. 20%), and a larger reduction in PTSD symptoms compared to those who received midazolam.

### Depression and comorbid symptom outcomes

Four studies examined the effects of ketamine on depression. Albott et al.^30^ observed that combat veterans showed reduced major depressive disorder symptoms following a high‐dose ketamine infusion. Similarly, Artin et al.^31^ reported that veterans with comorbid treatment‐resistant depression and PTSD receiving intranasal esketamine experienced a significant decrease in PHQ‐9 scores from 19.8 to 14.7 during the initial phase of treatment (*p* = 1 × 10^−6^). Notably, both depression and PTSD symptoms followed distinct trajectories of improvement. For example, depressive symptoms improved rapidly and then plateaued, whereas PTSD symptoms continued to improve throughout the treatment period.^31^ However, not all studies have shown consistent benefits for PTSD. Abdallah et al.^28^ observed no clear dose–response relationship for PTSD symptom reduction. Specifically, patients administered with the standard 0.5 mg/kg dose of ketamine demonstrated lower Montgomery–Asberg Depression Rating Scale (MADRS) scores both at 24 h post‐infusion and at the end of treatment.^28^

Additionally, secondary outcomes from Dadabayev et al.^23^ demonstrated that patients with comorbid PTSD and chronic pain experienced greater reductions in pain, as measured by VAS scores, compared to those with chronic pain alone on Day 7 (−30.61 vs. −14.28, *p* < 0.001). Collectively, these findings suggest that ketamine may exert therapeutic effects that extend beyond alleviation of PTSD symptoms, including improvements in comorbid depressive symptoms, although effects on PTSD itself are inconsistent across studies.^27^, ^28^

### Military and veteran populations: Descriptive findings

Several studies have been conducted in military‐affiliated or VA settings.^28^, ^30^, ^31^, ^32^ Veterans constituted a significant proportion of the study populations reviewed and were among the most frequently examined subgroups receiving ketamine interventions for PTSD. One study specifically targeted veterans with PTSD and MDD,^30^ while another recruited U.S. combat veterans for a prospective pre–post investigation.^32^ A retrospective case series also evaluated intranasal esketamine treatment among veterans receiving care in a VA neuromodulation clinic.^31^

Across these military‐focused studies, ketamine‐based interventions were associated with reductions in PTSD and depressive symptom severity. However, treatment effects varied by study design, with larger effects generally reported in open‐label and observational studies rather than in randomized‐controlled trials. In contrast, the largest multicenter RCT by Abdallah et al.,^28^ which included veterans and active‐duty service members, did not demonstrate significant superiority of ketamine over placebo for PTSD outcomes, although antidepressant effects were observed. Short‐term tolerability appeared acceptable under monitored conditions; however, the limited sample sizes, brief follow‐up, and incomplete safety reporting preclude firm conclusions regarding overall safety.

Despite the fact that military and veteran populations are clinically important and reported frequently in the current evidence base, the available studies do not establish differential efficacy compared with civilian populations. Given these factors, military and veteran samples should remain a research priority to better understand treatment outcomes and address the clinical challenges faced by these groups.

### Safety and tolerability

#### Dissociation and psychotomimetic effects

The most reported adverse effect across included studies was transient dissociation, which occurred during—or shortly after—ketamine administration. Most notably, Abdallah et al.^28^ reported increased dissociative symptoms, such as feelings of detachment, during ketamine administration, as measured by the Clinician‐Administered Dissociative States Scale (CADSS) (F [2,147] = 20.2; *p* < 0.0001). These symptoms resolved spontaneously during the recovery period without requiring further intervention.^28^ The highest CADSS scores were observed in the group receiving 1.0 mg/kg, demonstrating a dose‐dependent relationship.^29^ Dissociative effects were typically transient, lasting approximately two hours post‐infusion, and were not associated with psychotic or manic states.^27^, ^29^

#### Hemodynamic effects

Transient cardiovascular changes, including short‐term increases in blood pressure, were noted in several trials—particularly at higher doses—but were described as dose‐dependent, self‐limited, and not associated with serious or sustained cardiovascular events.^27^, ^29^

#### Common adverse effects

Additional adverse effects reported across studies included agitation, anxiety, irritability, constipation, nausea, dizziness, and fatigue. Repeated intravenous ketamine administration was reported to be generally well tolerated in two small studies.^27^, ^30^ Both ketamine and ketorolac were reported to be generally well tolerated following a single infusion.^23^ However, broad claims of safety should be interpreted in the context of the limited sample size and follow‐up durations of the included studies.

#### Cognitive effects

Neurocognitive function was evaluated across several time points, with findings showing no evidence of cognitive decline following six intravenous ketamine infusions over 12 days, including among participants with traumatic brain injury.^29^, ^33^ While this finding is promising, further neurocognitive research with longer follow‐up is required.

#### Serious adverse events and withdrawals

No serious adverse events were reported following ketamine administration, although safety conclusions are constrained by modest sample sizes and limited follow‐up. One participant withdrew from treatment due to acute adverse reactions.^23^ No reports of psychosis, mania, or persistent psychiatric deterioration were identified.

#### Abuse liability

Abuse potential or dependence liability was not formally evaluated in any of the reviewed studies. Given that ketamine is classified as a Schedule III controlled substance in the United States and that PTSD populations frequently have co‐occurring substance use disorders, this represents a considerable clinical gap.

#### Durability of treatment effect and follow‐up limitations

The long‐term efficacy of ketamine for PTSD remains unclear. Follow‐up periods across included studies ranged from 7 days^29^ to 8 weeks post‐final infusion.^30^ The longest available follow‐up was 90 days, where ketamine‐treated participants maintained significantly lower PTSD symptoms relative to midazolam.^15^ No included study provided data beyond the 90 days.

## DISCUSSION

### Principal findings

This systematic review analyzed eight studies investigating the effectiveness and safety of ketamine for PTSD. Although several studies reported reductions in PTSD symptom severity following ketamine administration, the overall evidence remains inconclusive. Notably, the largest randomized‐controlled trial did not demonstrate superiority of ketamine over placebo for the primary PTSD outcomes, whereas some of the largest reported treatment effects originated from small uncontrolled or open‐label studies that are more susceptible to placebo effects, expectancy effects, regression to the mean, and other sources of bias. Controlled studies produced inconsistent findings, and the available evidence therefore does not support a definitive conclusion regarding the efficacy of ketamine for PTSD. Small sample sizes, methodological heterogeneity, brief follow‐up, and incomplete safety reporting further limit interpretation. Findings from military and veteran samples were therefore summarized descriptively, as the available evidence did not permit formal subgroup analysis.

### Efficacy

The most prominent reductions in PTSD symptoms were reported in small uncontrolled or open‐label studies, including studies of treatment‐resistant military veterans. Although these studies reported large effect sizes—with some patients showing Cohen's d values reaching or exceeding 2.0—their findings should be interpreted with caution because the absence of control groups limits attribution of the observed changes specifically to ketamine. Rapid onset of effects, including symptom reductions within 24 h of the first infusion, was reported in some trials.^15^, ^29^ However, these encouraging findings were not consistently replicated in controlled studies. In the largest randomized‐controlled trial, Abdallah et al.^28^ found no statistically significant between‐group difference on the primary PTSD outcome measure. A statistically significant advantage was observed only in the lower‐dose arm (0.2 mg/kg) on the PCL‐5 at 4 weeks, while the 0.5 mg/kg dose—despite showing superiority for depressive symptoms—did not significantly differ from placebo for PTSD outcomes.^28^ The differing dose–response patterns observed for depressive and PTSD symptoms are nevertheless noteworthy and may have implications for understanding ketamine's mechanism of action and for dose selection in future trials. Baseline cognitive function has also been identified as a potential moderator of treatment response, with lower working memory capacity (*r* = −0.545, *p* = 0.036) and slower processing speed (*r* = 0.586, *p* = 0.028) associated with greater PTSD symptom improvement following ketamine.^30^ If replicated, these findings could help inform patient selection strategies. Taken together, however, the controlled evidence remains inconsistent and does not establish a reliable treatment effect of ketamine for PTSD.

### Safety

The available studies provide limited information regarding the short‐term safety and tolerability of ketamine under monitored conditions; however, small sample sizes, brief follow‐up, and incomplete adverse‐event reporting in four studies limit firm conclusions regarding its overall safety profile. Dissociation and psychotomimetic reactions during ketamine infusion were among the most frequently reported adverse events and generally resolved spontaneously after administration.^28^, ^29^ Hemodynamic monitoring, with beta‐blocker administration when clinically indicated, may therefore represent a feasible management strategy in ketamine treatment programs.

Furthermore, no cognitive decline was reported following repeated intravenous ketamine administration,^30^, ^33^ including protocols involving six infusions. However, these findings should not be interpreted as evidence that repeated ketamine administration is free of cognitive risk, because the available studies were small, had limited follow‐up, and were not specifically designed or powered to detect longer‐term neurocognitive adverse effects. Similarly, abuse liability and dependence potential were not formally evaluated in any included study. Therefore, their absence from the reported findings should not be interpreted as evidence of absence. This remains an important evidence gap, given Ketamine's Schedule III status and the elevated prevalence of substance use disorders in PTSD populations. Serious adverse events were also infrequently reported, but the available evidence is insufficient to exclude uncommon or delayed safety outcomes.

The current review was also unable to comprehensively determine whether the included trials assessed functional unblinding, defined as participants or assessors correctly inferring treatment allocation because of ketamine's recognizable dissociative and psychotomimetic effects. These effects can make effective blinding difficult,^15^, ^23^, ^34^ and failure to assess blinding integrity represents an important methodological limitation in ketamine trials for PTSD.^34^ Although none of the included studies formally assessed blinding integrity through treatment‐allocation guesses, Abdallah et al.^28^ and Feder et al.^15^ acknowledged the blinding integrity as a potential concern because participants receiving ketamine may recognize its characteristic subjective effects. This could, in turn, play a role in increasing expectancy‐related treatment effects and bias patient‐reported outcomes. Accordingly, expectancy effects cannot be excluded as contributors to the observed treatment benefits. Future trials should incorporate systematic assessments of blinding integrity, such as asking participants and assessors to guess treatment allocation after each session.

### Military and veteran populations: Descriptive findings

Several of the included studies were conducted in military or veteran populations, allowing descriptive consideration of ketamine use in these groups. However, no formal subgroup analysis or direct comparison between military/veteran and civilian populations was possible because of the small number of studies and substantial methodological heterogeneity. While the available studies suggest that ketamine administration is feasible and generally tolerable in monitored military and veteran settings,^28^, ^30^, ^31^ the current evidence base does not establish greater efficacy in these populations than in civilians. Further studies specifically designed to compare treatment outcomes across military, veteran, and civilian populations are warranted, particularly given the high burden of treatment‐resistant PTSD, psychiatric comorbidity, functional impairment, and suicide risk in military and veteran populations.

### Clinical implications

Ketamine is currently regarded as an investigational drug for the management of PTSD, as the available evidence is insufficient to support its routine or first‐line clinical use. Although the findings justify further controlled investigation, inconsistent efficacy results, limited long‐term follow‐up, incomplete adverse‐event reporting, and the absence of formal assessment of abuse liability and dependence substantially limit conclusions regarding the benefit–risk profile.

Any use of ketamine in clinical or research settings should therefore involve careful patient selection, including consideration of substance use history, cardiovascular risk, psychiatric comorbidity, and cognitive status. Monitoring of cardiovascular parameters, dissociative and psychotomimetic effects, and treatment outcomes is essential during administration. Because the durability of treatment effects remains uncertain, clinical use should also include structured follow‐ups and careful evaluation of any repeat‐dosing or re‐administration protocols.

Ketamine programs for patients with PTSD should operate within a framework that includes appropriate medical supervision, safeguards against misuse, and ongoing psychological assessments. Until larger, adequately controlled studies with longer follow‐up and more comprehensive safety assessment are available, ketamine should not be considered an established treatment for PTSD.

### Limitations

This review has numerous limitations that should be considered when interpreting the findings. First, the evidence base was limited, with only eight studies meeting the inclusion criteria and sample sizes ranging from 15 to 158 participants, which may have been insufficient to detect modest treatment effects or rare safety signals. Next, significant variations were noted across studies in the formulation, dose, route, timing, and treatment duration, making a pooled meta‐analysis unsuitable and direct cross‐study comparisons difficult. Beyond the aforementioned methodological heterogeneity, variability in study design was also evident, ranging from small open‐label trials to larger RCTs. This, in turn, increased the risk of bias, particularly in uncontrolled trials where placebo response, expectancy effects, and regression to the mean were plausible. A further concern is functional unblinding, as ketamine's recognizable dissociative and psychotomimetic effects may have revealed treatment allocation, potentially increasing expectancy bias, particularly for patient‐reported outcomes such as the PCL‐5 and IES‐R. Although active comparators may improve masking, the included trials did not formally assess blinding integrity; therefore, the extent to which functional unblinding influenced the observed treatment effects remains uncertain. Another limitation is the brief follow‐up period, which did not exceed 90 days. Consequently, the durability of ketamine's effects and the risk of relapse remain unclear. Incomplete adverse‐event reporting represents a further limitation, as four studies did not provide complete safety data, restricting conclusions regarding ketamine's overall safety profile. Interpretability of the findings was further limited by the lack of PTSD diagnostic criteria specification noted in one study. In addition, none of the included studies formally evaluated abuse liability or dependence potential. Furthermore, given the absence of meta‐analysis and the small number of included studies, formal testing for statistical publication bias was not possible. Finally, although supervisory verification was performed, the review was not conducted independently in duplicate, which may have increased the risk of selection, extraction, and assessment bias.

## CONCLUSION

This review synthesized evidence from eight eligible studies examining the effects of ketamine and esketamine on PTSD, with a focus on military and veteran populations. Despite the fact that current evidence suggests that ketamine has been associated with short‐term reduction in PTSD symptoms in some studies—with small advantages over control conditions that appear to diminish over time—the controlled evidence remains inconsistent. Therefore, the present findings are to be considered preliminary and limited by small sample sizes, heterogeneous designs, short follow‐up, and inconsistent safety and tolerability reporting. Given these mixed and still‐evolving outcomes, ketamine and esketamine should be considered investigational options for PTSD, used only in structured clinical or research settings and integrated with trauma‐focused psychotherapy rather than as stand‐alone pharmacotherapy. Future trials should use adequately powered randomized designs, longer follow‐up, standardized safety reporting, formal assessment of abuse liability, and intentional enrollment of military and veteran populations.

## AUTHOR CONTRIBUTIONS

Mary Ivasyutyn contributed to conceptualization, literature search, study screening, data extraction, quality assessment, narrative synthesis, drafting of the manuscript, table preparation, and manuscript revisions. Saajan Bhakta contributed to conceptualization, methodological guidance, supervision, critical review of the manuscript, interpretation of findings, manuscript revision, and final approval of the manuscript. Both authors reviewed and approved the final version of the manuscript and agree to be accountable for the accuracy and integrity of the work.

## CONFLICT OF INTEREST STATEMENT

The authors declare no conflicts of interest.

## ETHICS APPROVAL STATEMENT

Ethics approval was not required because this study is a systematic review of previously published literature and did not involve direct participation of human subjects or access to identifiable private information.

## PATIENT CONSENT STATEMENT

Not applicable.

## PERMISSION TO REPRODUCE MATERIAL FROM OTHER SOURCES

Not applicable. No copyrighted material requiring permission was reproduced in this manuscript. If any figure, table, or adapted material from another source is used, this statement will need to be revised and appropriate permission obtained.

## CLINICAL TRIAL REGISTRATION

Not applicable. This study is a systematic review and was not a clinical trial.

## SYSTEMATIC REVIEW REGISTRATION

This systematic review was registered in PROSPERO under registration number CRD420261401140. Registration occurred after the review process had begun and is therefore acknowledged as a methodological limitation.

## Data Availability

Data sharing is not applicable to this article because no new data sets were generated or analyzed. This systematic review synthesized findings from previously published studies.
